# Changes in Ganglion Cell Complex and Peripapillary Retinal Nerve Fiber Layer after Femtosecond Laser-Assisted Cataract Surgery Compared to Manual Phacoemulsification in Patients Receiving a Trifocal Intraocular Lens

**DOI:** 10.1155/2020/8626495

**Published:** 2020-08-06

**Authors:** Carmen Sánchez-Sánchez, Laureano A. Rementería-Capelo, Virginia Carrillo, Juan Pérez-Lanzac, Inés Contreras

**Affiliations:** ^1^Clínica Rementería, Madrid, Spain; ^2^Hospital Universitario Ramón y Cajal, Instituto Ramón y Cajal de Investigaciones Sanitarias (IRYCIS), Madrid, Spain

## Abstract

**Introduction:**

During femtosecond laser-assisted cataract surgery (FLACS), there is a significant increase in intraocular pressure, which might lead to ganglion cell damage. We aimed to determine whether there were differences in the changes produced in the ganglion cell complex (GCC) and peripapillary retinal nerve fiber layer (pRNFL) thickness, as evaluated with optical coherence tomography (OCT), between phacoemulsification and FLACS, after implantation of a trifocal intraocular lens (IOL).

**Methods:**

Patients with no coexistent pathologies undergoing cataract surgery with implantation of a PanOptix IOL were explored with the Cirrus-OCT before and three months after surgery. GCC values were obtained from the built-in software. The differences between pre- and postoperative GCC and pRNFL thicknesses after phacoemulsification were compared to differences after FLACS.

**Results:**

A total of 171 eyes were included, 74 undergoing FLACS and 97 phacoemulsification. For both groups, there was a statistically significant increase in GCC values after cataract surgery, except for the inferior and inferonasal sectors. There were no statistically significant differences between FLACS and phacoemulsification. Mean change in average GCC and minimum GCC were 1.08 ± 1.40 *µ*m (range −1 to +6 *µ*m) and 1.69 ± 2.54 *µ*m (range −3 to +11 *µ*m) after FLACS and 0.99 ± 1.67 *µ*m (range −5 to +6 *µ*m) and 2.02 ± 3.54 *µ*m (−6 to +18 *µ*m) after phacoemulsification. These values are similar to those previously reported after phacoemulsification with monofocal IOL implantation. No significant changes after surgery were detected for the pRNFL, with no differences between groups. *Discussion*. There were no differences in the changes produced by FLACS and phacoemulsification in either GCC or pRNFL values. Although mean change was small, the range of variation was wide. Therefore, it is necessary to establish a new baseline for GCC and pRNFL thicknesses after cataract surgery in order to monitor any subsequent changes.

## 1. Introduction

Optical coherence tomography (OCT) has become one of the mainstays in the evaluation of ophthalmic patients. In the management of glaucoma, detecting structural change over time in the peripapillary retinal nerve fiber layer (pRNFL) and in the ganglion cell complex (GCC) is useful for both the initial diagnosis and detecting progression. Many patients show structural changes in the absence of visual field deterioration, providing the opportunity to start or increase treatment before the onset of permanent visual damage [1, 2]. Detecting change over time has advantages over comparing a single scan with a normative database, due to the fact that “normal” values vary widely between different subjects. Thus, it is possible for a patient to suffer significant neural loss before being deemed outside normal limits [3]. Several studies have reported that GCC and pRNFL thickness parameters significantly increase after cataract surgery [4–7] with a tendency to decrease with time [8], although without reaching preoperative levels [9, 10].

Nowadays, femtosecond laser-assisted cataract surgery (FLACS) is expanding. It is well known that cataract surgery leads to a transient intraocular pressure (IOP) elevation in the early postoperative period [11]. In addition, the femtosecond procedure leads to a significant increase in IOP: experiments in ex-vivo porcine models have reported that the IOP increases during the treatment steps to up to 48.41 ± 6.80 mmHg with the Catalys platform [12], 52.00 ± 6.35 mmHg with the Femto LDV Z8 [13], and 77.01 ± 5.88 mmHg with the Victus platform [14]. An in vivo trial with IOP measurement with an I-Care tonometer found that IOP rose from a preoperative mean of 13.8 ± 0.4 mmHg to 24.2 ± 1.4 mmHg one minute after docking release in patients undergoing FLACS with a LenSx system [15]. In another in vivo trial, IOP rose to 42.1 ± 10.8 mm Hg during the suction phase with the Victus platform [16]. There is some concern that these IOP spikes, in elderly patients with more vulnerable optic nerves, might lead to retinal nerve fiber damage. Animal models have found that even transient ocular hypertension may lead to early changes in the structure and function of various retinal ganglion cell types [17].

The purpose of this study was to evaluate the changes produced in GCC values after FLACS and compare them with changes produced after “classic” phacoemulsification, with the implantation of a trifocal intraocular lens (IOL), in order to determine whether IOP increase during the femtosecond procedure might lead to ganglion cell damage, reflected in OCT measurements.

## 2. Methods

All patients who underwent cataract surgery with implantation of a trifocal IOL AcrySof IQ PanOptix^TM^ between January 2017 and June 2018 in our center were considered for inclusion. At the one-month postoperative visit, the nature of the study was explained and all those patients who agreed to participate and signed the informed consent were scheduled for a visit three months after surgery. The study was approved by our ethics committee and followed the tenets of the declaration of Helsinki.

Inclusion criteria were age over 18 years, preoperative OCT with signal strength ≥6, and uneventful cataract surgery with Panoptix IOL implantation. Exclusion criteria were presence of ocular pathologies, amblyopia, postoperative complications, and suboptimal segmentation of ganglion cell complex. Only one eye per subject was included in the study. The right eye was chosen unless preoperative signal strength was below required or there was incorrect segmentation, in which case the left eye was chosen.

Candidates to cataract surgery underwent a comprehensive preoperative evaluation including distance-corrected visual acuity, slit-lamp examination, tonometry, corneal topography (Pentacam HR model 70,900, Oculus, Germany), endothelial cell count (CEM-530 specular biomicroscope, NIDEK CO, LDT, Japan), biometry (IOLMaster, Carl Zeiss Meditec, Germany), fundus evaluation after pharmacological mydriasis, and optic nerve head and macular OCT examination.

**Figure 1:**
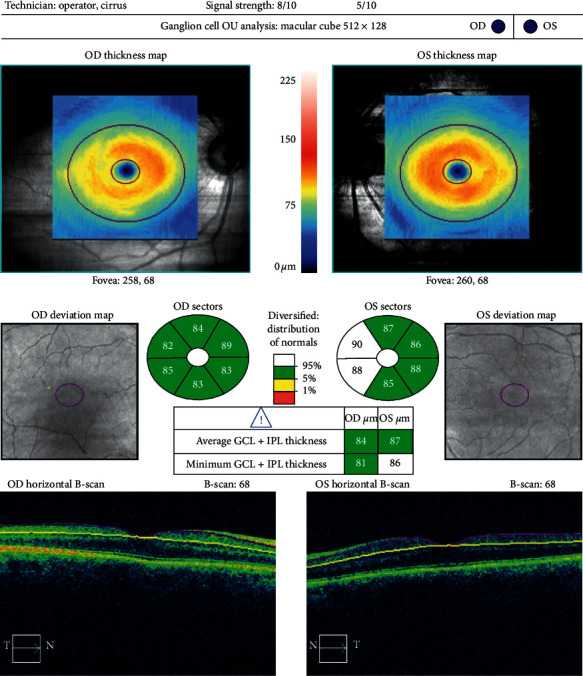
Preoperative Ganglion Cell Analysis of a 67-year-old male.

**Figure 2:**
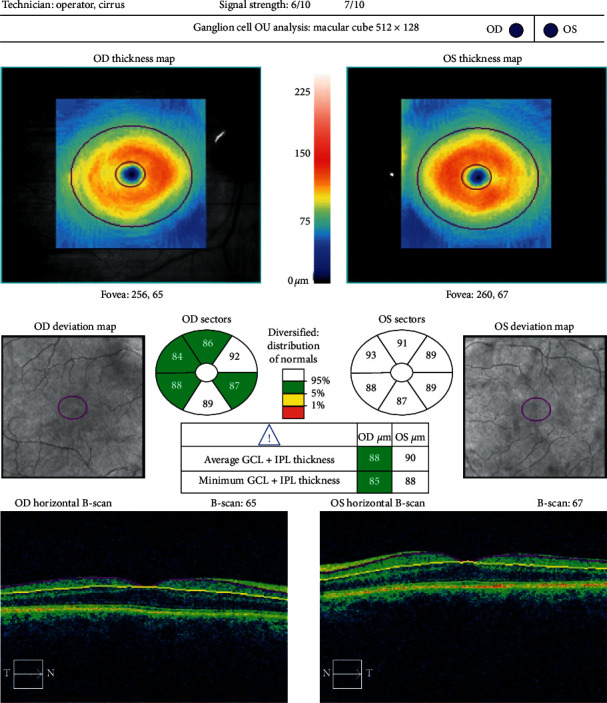
Postoperative Ganglion Cell Analysis of the same patient shown in Figure 1.

**Figure 3:**
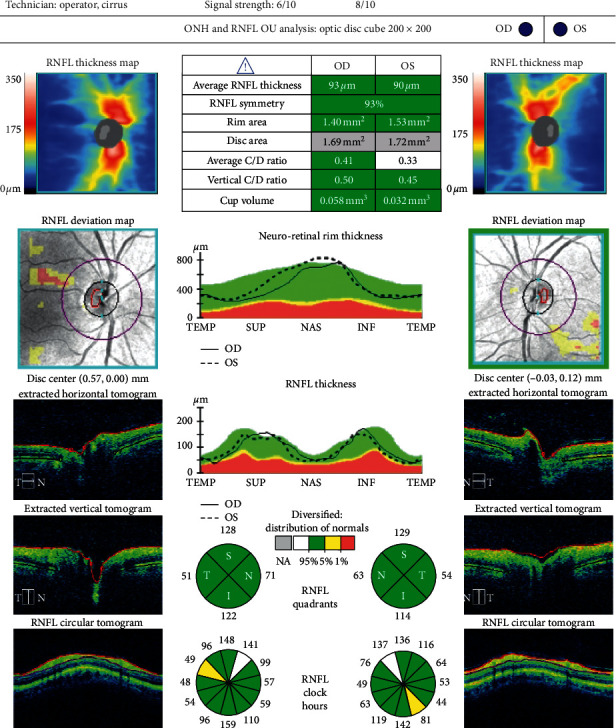
Preoperative retinal nerve fiber layer (RNFL) analysis of a 74 year-old male.

**Figure 4:**
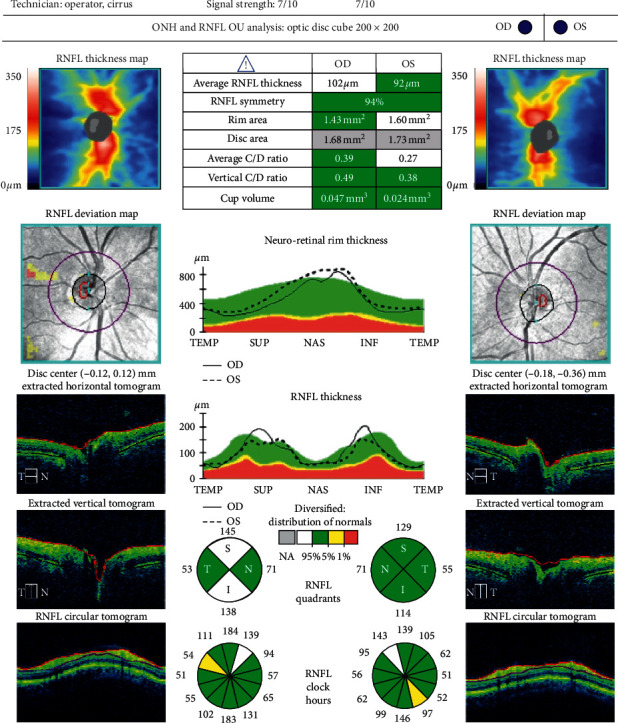
Postoperative retinal nerve fiber layer (RNFL) analysis of the same patient shown in Figure 3.

Optical coherence tomography measurements were performed with the Cirrus HD OCT imaging system (Carl Zeiss Meditec, USA), before and three months after cataract surgery. For macular examination, the macular cube 512 × 128 acquisition protocol was used. This protocol generates a cube through a 6 mm square grid of 128 B-scans, each composed of 512 A-scans. The built-in software identifies the outer boundaries of the RNFL and the inner plexiform layer. The difference between the RNFL and the inner plexiform layer outer boundary segmentations yields the combined thickness of the retinal ganglion cell/inner plexiform layer, which we will refer to as GCC. The Ganglion Cell Analysis algorithm provides GCC measurements in six wedge-shaped sectors centered on the fovea. It also gives information on the average and minimum GCC thickness for each eye and compares these figures with a normative database. Examples of a pre- and postoperative analysis are shown in Figures 1 and 2. The Optic Disc Cube 200 × 200 was used to capture images and measure pRNFL thickness. Examples of a pre- and postoperative analysis are shown in Figures 3 and 4.

FLACS was performed under topical anesthesia with the LenSx platform (Alcon Laboratories, USA), with the Contact lens SoftFit™ interface. The soft hydrogel contact lens matches the corneal curvature with minimal distortions, helping to decrease the pressure necessary to fix the eyeball. The main corneal incision was fixed at 135 ° with a width of 2.3 mm and the sideport at 60° with a width of 1 mm. The capsulotomy had a diameter of 5 mm and a combined radial and cylinder pattern was employed for lens fragmentation. Surgery was completed with the Centurion® Vision system (Alcon Laboratories, USA). The Panoptix IOL was injected in the bag. For patients undergoing “classic” phacoemulsification, surgery was performed through a 2.2 mm clear corneal incision, with a Stop and Chop technique. Intracameral cefuroxime was used at the end of all procedures as prophylaxis for endophthalmitis.

Statistical analysis was performed with SPSS software (version 20.0, IBM SPSS, USA). The Kolmogorov–Smirnov test was used to evaluate the normal distribution of measured data. When parametric analysis was possible, Student's *t* test for paired data was employed to compare pre- and postoperative values for both groups and Student's *t* test for unpaired data was used for evaluating differences between the FLACS and phacoemulsification groups. The Bonferroni correction was applied due to the multiple comparisons performed, so that significance was set at *p* < 0.0015.

## 3. Results

A total of 171 eyes of 171 patients were included in the study. Of these, 74 eyes underwent FLACS (FLACS group) and 97 eyes “classic” phacoemulsification (Phaco group). There was a higher percentage of women in the FLACS group (58/74 eyes, 78.4%) compared to the Phaco group (67/97, 69.1%), although the difference was not statistically significant, *p*=0.223. Mean age was similar in both groups: 67.12 ± 7.80 years for FLACS versus 67.74 ± 7.93 for the Phaco group.  Table 1 shows the preoperative characteristics of the eyes included in the study.

Tables 2 and 3 show the pre- and postoperative values of the GCC for the Phaco and FLACS groups, respectively. For both groups, there was a statistically significant increase in all GCC values except for the inferior and inferonasal sectors. There were no statistically significant differences in GCC changes between the FLACS and Phaco groups (Table 4). As regards pRNFL thickness, there were no statistically significant changes with cataract surgery, neither with FLACS nor classic phacoemulsification (Tables 5 and 6).

## 4. Discussion

Optical coherence tomography examination of the optic nerve head and the macula is nowadays performed almost systematically as part of ophthalmic evaluations. Measurements of the pRNFL and the GCC thicknesses are used to diagnose glaucoma and to monitor the course of the disease. Several studies have reported that cataract surgery leads to changes in the GCC and pRNFL thickness values [4–10] and these changes must be taken into account for patient follow-up. However, to the best of our knowledge, no study has directly compared the effect of FLACS with “classic” phacoemulsification on GCC values. Since FLACS has been shown to be associated with a significant, although transient, increase in IOP, this comparison might detect differences in postoperative GCC and pRNFL thickness due to ganglion cell damage.

In our study, we found no differences in the changes produced by FLACS and “classic” phacoemulsification in GCC values. The mean increase in average GCC was 1.08 ± 1.40 *µ*m for the FLACS group compared to 0.99 ± 1.67 *µ*m for the Phaco group. This change in average GCC value is comparable to those reported in other studies evaluating changes after classic phacoemulsification with Cirrus-OCT, with increases ranging from 0.57 to 4.2 *µ*m [4, 7, 8]. However, although the mean change across the whole study group is small, the range of variation we found was wide: for a given individual, average GCC can vary as much as 6 *µ*m, minimum GCC as much as 18 *µ*m, and the value for some sectors as much as 17 µm. Therefore, it is imperative to establish a new baseline for GCC after cataract surgery in order to monitor any subsequent changes.

As regards changes in average pRNFL thickness, a mean increase of between 2.11 µm and 5.63 *µ*m has been reported after classic phacoemulsification using a Cirrus-OCT [4, 5, 7, 8]. In our study, we found that average pRNFL did not change significantly after cataract surgery, with no differences between the phaco and FLACS groups. This might be due to the larger number of patients included in our study, which might tend to compensate differences. Regarding the specific effect of FLACS, Reñones et al. reported a mean increase of 2.16 *µ*m in average pRNFL as evaluated with Spectralis-OCT, 6 months after surgery [18]. Their study did not include a control group undergoing classic phacoemulsification. Although comparisons must be made with caution, since it has been reported that there are differences in RNFL measurements between devices [5], it seems that pRNFL change after FLACS is within the values reported for phacoemulsification.

The reason for the changes in OCT measurements after cataract surgery is not completely clear. It seems that it may be due to a combination of several factors, including the inflammatory effect of the procedure, a decrease in the optical density of the lens, the change in refraction after surgery, and the optical properties of the IOL [5, 6]. The implantation of trifocal IOLs is steadily increasing and it is necessary to determine how they might affect OCT measurements. García-Bello et al. [19] found that the implantation of a trifocal diffractive IOL (AT LISA® Tri 839 MP, Zeiss) produced a higher difference in average pRNFL measurements than the implantation of a monofocal IOL (CT ASPHINA 409 M/MP): 7.29 ± 10.51 *µ*m versus 1.96 ± 2.90 *µ*m, respectively, *p*=0.017. However, the results of this study must be taken cautiously. Only 25 eyes were included per group and the trifocal group had a low preoperative average pRNFL with a wide standard deviation: 79.67 ± 15.21 *µ*m versus 100.35 ± 6.44 *µ*m for the monofocal group [19]. Thus, the greater difference found for trifocal lens might have been due to inaccurate preoperative measurements. The same group has published another study comparing the AT LISA tri839MP and the FineVision IOL (PhysIOL, Belgium), another trifocal IOL, with 24 patients per group [20]. In this study, average pRNFL thickness increased in the Finevision group from 88.06 ± 13.65 *µ*m to 92.04 ± 12.78 and in the AT LISA group from 82.39 ± 15.14 to 86.88 ± 10.95 *µ*m. These results are within the changes reported for monofocal IOLs. In our study, all patients received a Panoptix trifocal IOL; again, we believe the lack of a significant difference in mean average pRNFL thickness might be due to the larger number of patients included. However, as is the case for CCG measurements, although there is no significant difference in the mean average pRNFL, average pRNFL in a given patient might change as much as 10 *µ*m and therefore it is necessary to acquire a new baseline after surgery for further follow-up.

The main limitation of our study was that patients were not randomized to FLACS or classic phacoemulsification. However, the groups had similar baseline characteristics. On the other hand, two of the strengths of our study are the large number of patients included and the fact that only one eye per patient was analyzed.

In summary, we found that both FLACS and classic phacoemulsification lead to a small but statistically significant increase in most GCC measurements with Cirrus-OCT. No difference was found for average pRNFL measurements. There were no differences in the changes produced between FLACS and classic phacoemulsification. Although mean changes are low, individual variations mean that a new baseline should be acquired after surgery.

## Figures and Tables

**Table 1:** Preoperative characteristics of the eyes included in the study.

	FLACS	Phaco	*P*
LogMAR visual acuity	0.14 ± 0.21	0.15 ± 0.20	0.868
Pachymetry (*µ*m)	554 ± 31.26	544 ± 36.47	0.057
Endothelial cell count (cells/mm^2^)	2400 ± 322.53	2435 ± 334.93	0.504
Axial length (mm)	23.53 ± 1.35	23.43 ± 1.39	0.643
Anterior chamber depth (mm)	3.18 ± 0.40	3.11 ± 0.36	0.255

FLACS: femtosecond laser-assisted cataract surgery. Phaco: phacoemulsification. Values provided are mean ± standard deviation.

**Table 2:** Pre- and postoperative values of ganglion cell complex (GCC) for classic phacoemulsification cataract surgery.

	Preoperative	Postoperative	*P*
Average GCC (*µ*m)	79.88 ± 6.55	80.87 ± 6.66	**<0.001**
Minimum GCC (*µ*m)	76.56 ± 7.45	78.58 ± 6.92	**<0.001**
Inferotemporal GCC (*µ*m)	80.73 ± 6.10	81.70 ± 6.24	**<0.001**
Inferior GCC (*µ*m)	79.23 ± 6.54	79.88 ± 6.68	0.027
Inferonasal GCC (*µ*m)	79.81 ± 7.49	80.57 ± 7.83	0.012
Superonasal GCC (*µ*m)	80.93 ± 7.97	82.33 ± 7.87	**<0.001**
Superior GCC (*µ*m)	79.94 ± 7.38	80.99 ± 7.39	**<0.001**
Superotemporal GCC (*µ*m)	78.69 ± 6.59	79.70 ± 6.71	**<0.001**

Values provided are mean ± standard deviation. Values in bold are those considered statistically significant taking into account the Bonferroni correction.

**Table 3:** Pre- and postoperative values of ganglion cell complex (GCC) for femtosecond laser-assisted cataract surgery.

	Preoperative	Postoperative	*P*
Average GCC (*µ*m)	79.16 ± 6.35	80.24 ± 6.33	**<0.001**
Minimum GCC (*µ*m)	76.57 ± 6.68	78.26 ± 6.65	**<0.001**
Inferotemporal GCC (*µ*m)	79.97 ± 6.97	80.97 ± 6.74	**0.001**
Inferior GCC (*µ*m)	77.97 ± 6.88	78.41 ± 7.32	0.096
Inferonasal GCC (*µ*m)	78.57 ± 7.43	79.57 ± 7.55	0.004
Superonasal GCC (*µ*m)	80.31 ± 7.03	81.89 ± 7.49	**<0.001**
Superior GCC (*µ*m)	79.71 ± 6.31	81.12 ± 6.10	**<0.001**
Superotemporal GCC (*µ*m)	78.13 ± 6.40	79.65 ± 5.96	**<0.001**

Values provided are mean ± standard deviation. Values in bold are those considered statistically significant taking into account the Bonferroni correction.

**Table 4:** Differences in pre- and postoperative ganglion cell complex measurements for femtosecond laser-assisted cataract surgery compared with classic phacoemulsification.

	FLACS	Phaco	*P*
Average GCC (*µ*m)	1.08 ± 1.40 −1 to +6	0.99 ± 1.67 −5 to +6	0.707
Minimum GCC (*µ*m)	1.69 ± 2.54 −3 to +11	2.02 ± 3.54 −6 to +18	0.496
Inferotemporal GCC (*µ*m)	1.01 ± 2.49 −10 to + 10	0.97 ± 2.11 −8 to +7	0.900
Inferior GCC (*µ*m)	0.43 ± 2.20 −6 to +5	0.65 ± 2.84 −7 to +11	0.587
Inferonasal GCC (*µ*m)	1.00 ± 2.90 −5 to + 17	0.75 ± 2.90 −5 to +15	0.581
Superonasal GCC (*µ*m)	1.58 ± 2.81 −4 to + 11	1.40 ± 2.66 −4 to +12	0.671
Superior GCC (*µ*m)	1.40 ± 2.53 −5 to +9	1.05 ± 2.59 −7 to +8	0.372
Superotemporal GCC (*µ*m)	1.51 ± 2.14 −6 to +6	1.01 ± 2.51 −5 to +10	0.169

FLACS: femtosecond laser-assisted cataract surgery. Phaco: phacoemulsification.

**Table 5:** Pre- and postoperative values of central macular thickness, macular volume, and peripapillary retinal nerve fiber layer (pRNFL) thickness for femtosecond laser-assisted cataract surgery and “classic” phacoemulsification.

	Preoperative	Postoperative	*P*
*FLACS*			
Central retinal thickness (*µ*m)	259.23 ± 19.70	264.57 ± 20.80	**<0.001**
Macular volume (mm^3^)	9.99 ± 0.50	10.14 ± 0.47	**<0.001**
Average pRNFL thickness (*µ*m)	90.22 ± 8.99	91.01 ± 9.90	0.019
*Phaco*			
Central retinal thickness (*µ*m)	260.44 ± 22.23	264.61 ± 22.02	**<0.001**
Macular volume (mm^3^)	10.01 ± 0.52	10.21 ± 0.61	**0.001**
Average pRNFL thickness (*µ*m)	91.16 ± 8.77	91.13 ± 8.22	0.696

FLACS: femtosecond laser-assisted cataract surgery. Phac: phacoemulsification. Values provided are mean ± standard deviation. Values in bold are those considered statistically significant taking into account the Bonferroni correction.

**Table 6:** Differences in pre- and postoperative values of central macular thickness, macular volume, and peripapillary retinal nerve fiber layer (pRNFL) thickness for femtosecond laser-assisted cataract surgery compared with “classic” phacoemulsification.

	FLACS	Phaco	*P*
Central retinal thickness (*µ*m)	5.34 ± 10.44 −8 to +82	4.30 ± 6.76 −35 to +21	0.109
Macular volume (mm^3^)	0.14 ± 0.25 −0.70 to +1.10	0.12 ± 0.33 −1.50 to +1.10	0.435
Average pRNFL thickness (*µ*m)	0.80 ± 3.70 −9 to +10	0.05 ± 3.89 −9 to +9	0.559

FLACS: femtosecond laser-assisted cataract surgery. Phaco: phacoemulsification.

## Data Availability

The dataset analyzed during the current study is available from the corresponding author on reasonable request.
